# ASSOCIATION BETWEEN DIET AND RETINAL IMAGING MARKER IN THE PREVENT DEMENTIA STUDY

**DOI:** 10.1093/geroni/igae098.3610

**Published:** 2024-12-31

**Authors:** Sarah Gregory, Samuel Gibbon, Thomas MacGillivray, Graciela Muniz Terrera

**Affiliations:** University of Edinburgh, Edinburgh, Scotland, United Kingdom; University of Edinburgh, Edinburgh, Scotland, United Kingdom; University of Edinburgh, Edinburgh, Scotland, United Kingdom; Ohio University, Athens, Ohio, United States

## Abstract

Eating a healthy diet is associated with a lower risk of neurodegenerative diseases, such as Alzheimer’s disease (AD). Identifying associations between diet and AD biomarkers is key to understanding and identifying critical periods for interventions. The retina provides a unique, non-invasive, window into brain health. This study explored cross-sectional and longitudinal associations between diet and retinal imaging markers in the PREVENT Dementia cohort (UK/Ireland). We calculated a Mediterranean diet (MedDiet) adherence score (Pyramid) for each participant and tested for associations with measures of retinal vessel morphology (CRAE Hubbard Zone C (HZC) and artery tortuosity) using generalised additive models due to abnormally distributed residuals. Additionally, we tested for associations with dietary intake of manganese. All analyses were completed cross-sectionally and then longitudinally in the participants with follow-up data (data collection ongoing). In a preliminary study, we included 86 participants, mean age 50.87 (5.63) years, majority female (57%), with 31 APOEe4 carriers (36%). Participants had a moderate mean Pyramid score (8.16 (SD = 1.40)), which was comparable to scores in other UK cohorts, and a dietary intake of 4.77 mg (SD = 3.05) manganese. There was evidence of an association between higher dietary manganese intake and lower CRAE HZC density in the right eye. There was no evidence of associations between the Pyramid score in either eye or dietary manganese in the left eye, and no signs of longitudinal associations (n=47). Further research is needed to explore any these analyses in a larger sample, and expand with other variables.

